# Excess deaths in treatment-resistant depression

**DOI:** 10.1177/20451253211006508

**Published:** 2021-04-12

**Authors:** Philip Brenner, Johan Reutfors, Michel Nijs, Therese M-L Andersson

**Affiliations:** Centre for Pharmacoepidemiology, Department of Medicine Solna, Karolinska Institutet, 171 77 Stockholm, Sweden; Centre for Pharmacoepidemiology, Department of Medicine Solna, Karolinska Institutet, Stockholm, Sweden; Janssen Global Services, Titusville, NJ, USA; Department of Medical Epidemiology and Biostatistics, Karolinska Institutet, Stockholm, Sweden

**Keywords:** depression, epidemiology, major depressive disorder, mortality, suicide, treatment-resistant

## Abstract

**Background:**

Patients with treatment-resistant depression (TRD) have an increased mortality risk compared with other patients with depression, but it is not known how this translates into absolute numbers of excess deaths.

**Methods:**

Swedish national registers were used to identify a cohort of 118,774 antidepressant initiators 18–69 years old with a depression diagnosis. Patients who initiated a third consecutive treatment trial were classified as having TRD. Flexible parametric survival models were used to estimate the mortality risk due to all causes and external causes (suicides and accidents), comparing TRD patients with patients with other depression while adjusting for clinical and sociodemographic covariates and including interactions with TRD, age, and Charlson comorbidity index (CCI) for a number of somatic comorbidities. Standardized survival was estimated, as were numbers of excess deaths among TRD patients within each age and comorbidity category.

**Results:**

Compared with the mortality risk of other depressed patients, patients with TRD experienced excess deaths in most age and comorbidity categories in the range of 7–16 deaths per 1000 patients during 5 years. Highest numbers for all-cause excess deaths were found among patients 18–29 years old with CCI 1, where 16 [95% confidence interval 5–28] of the expected 37 [25–48] deaths per 1000 patients were excess deaths. The majority of the excess deaths were due to external causes.

**Conclusion:**

Patients with TRD experience significant numbers of excess deaths compared with other patients with depression.

## Background

Several definitions of treatment-resistant depression (TRD) have been proposed, most of which are based on the concept of not achieving adequate response despite two separate, adequate treatment trials.^1,2^ TRD is common, with rates among patients initiating treatment for depression estimated at up to 50% in clinical cohorts,^3,4^ and in register based studies from 7% to 29%, depending on the method and definition used.^5,6^

Compared with other patients with depression, those with TRD may experience a greater risk of negative outcomes, including somatic and psychiatric comorbidities, poorer social outcomes, and lower quality of life.^7,8^ We recently found that patients fulfilling a pharmacoepidemiological definition of TRD had an increased mortality rate compared with other patients with depression, with a hazard ratio (HR) of 1.35, while the HR was 1.97 when only counting mortality from external causes, i.e., suicides and accidents.^9^

Measures of relative risks, such as HRs and incidence rate ratios, are commonly used for comparisons between groups. However, they are not always translatable to absolute numbers, and they seldom communicate an intuitive message of the actual impact that a condition, or preventive measure, may have on mortality. In our previous study, HRs comparing mortality among the TRD group with other depressed patients were higher in younger age groups and in groups without comorbidities.^9^ However, a high relative effect may not necessarily mean that the absolute effect is highest in these groups.

An alternative way of illustrating mortality differences is the number of deaths that count as excess deaths within a specific time period (e.g., 5 years) in a group of patients, compared with that group if it were to experience the same mortality as a comparison group. This may also be a relevant measure for patients as well as for clinicians and decision makers.^10^ Attempts to quantify the excess mortality from depression as number of deaths have been made, for example, in a recent study where 3.5% of deaths in the general population in the United States (US) were deemed attributable to anxiety and depression^11^; however, this measure does not convey excess deaths within the depressed population and does not take excess deaths in treatment-resistant patients into account.

Building on our previous investigation of the relative mortality risk associated with TRD, the purpose of this study was to estimate the absolute effect, that is, numbers of excess deaths among patients with TRD compared with other patients with depression, by using a cohort of patients with depression identified in nationwide Swedish registers. This is presented by age- and comorbidity groups.

## Material and methods

### Data sources and study population

As previously described,^9^ we used a combination of Swedish national registers to identify the study cohort, covariates, and outcomes. All patients 18–69 years old who filled a prescription for an antidepressant drug (any drug with an ATC-code starting with N06A) in Sweden between 1 July 2006 and 31 December 2014, without any previous antidepressant prescription for 180 days, were identified through The Prescribed Drug Register (PDR),^12^ which contains data on all prescribed and dispensed prescriptions in Sweden since 1 July 2005. Among these, patients who had a diagnosis of depression (ICD-10 F32, F33, or F34) in the National Patient Register (NPR) within a time interval of 30 days before, and up to 365 days after, the filled prescription were included in the depression cohort.^13^ The NPR contains data on all diagnoses and procedures during in-patient care and specialized out-patient care – excluding primary care – according to the International Statistical Classification of Diagnoses, 7th–10th editions (ICD 7–10) with complete national coverage for in-patient care since 1987, and for out-patient care since 2001. Exclusion criteria were fills of lithium (N05AN01), antipsychotics (N05A, except for lithium), and the anticonvulsants valproate (N03AG01), lamotrigine (N03AX09), and carbamazepine (N03AF01) during 180 days before the first antidepressant prescription, and also those with procedure codes for electroconvulsive therapy (ECT; DA006, DA024-25) or repetitive transcranial magnetic stimulation (rTMS; DA022, DU050). Patients with a previous history of psychosis (ICD-10 F20–F29), mania (F30), bipolar disorder (F31), or dementia (F00–F03) were also excluded, as were patients who were not residents in Sweden for the full 180 days according to the Total Population Register before the first antidepressant prescription filling.^14^ The index date was defined as the date when both the criteria of a prescription and a diagnosis were fulfilled. Patients could enter the study at any time during the study period when inclusion criteria were met.

### Definition of TRD

Patients were classified with TRD from the starting date of a third treatment trial for depression after having undergone two adequate trials. An adequate treatment trial was defined as lasting for at least 28 days. Subsequent treatment trials after the first dispensed antidepressant trial had to include at least one fill of a different antidepressant (as monotherapy or in combination with the previous one), add-on medication to the antidepressant (antipsychotics or anticonvulsants), or administration of ECT or rTMS. Durations of trials were estimated by semi-manually reading prescription dosage texts and taking into account the number of tablets dispensed at each fill, and by action codes for ECT/rTMS in the NPR. Treatment gaps of >28 days were not allowed in order to emulate consecutive treatment trials. Distribution of medication/treatments used for the third treatment trial is shown in Supplemental Table S1.

### Outcomes

The main outcomes were all-cause mortality and death from external causes (including suicides and accidents; ICD codes V01–Y98) as registered in the Cause of Death Register.^15^

### Covariates

The following covariates were added to the patients in the cohort: sex, age, history of depression before or within the last 5 years, self-harm (ICD-10: X60–X84 and Y10–Y34), substance use disorders [ICD-10: F10–16 and F18–19, or prescriptions of sublingual buprenorphine (ATC: N07BC01/ N07BC51), methadone (ATC: N07BC02), disulfiram (ATC: N07BB01), acamprosate (ATC: N07BB03), naltrexone (ATC: N07BB04), or nalmefen (ATC: N07BB05)], and 5-year history of other psychiatric comorbidity (anxiety disorder, attention deficit hyperactivity disorder, autism spectrum disorder, eating disorder, personality disorder; ICD-10: F40–F42, F50, F60–F61, F84.0–1, F84.5, and F90). A Charlson comorbidity index (CCI) was constructed from diagnoses in the NPR categorized as 17 major comorbidity groups adjusted for ICD-10 diagnostic coding.^16,17^ The CCI is a method of categorizing comorbidities of patients based on ICD diagnosis codes, and weighs comorbidities based on a predefined adjusted risk of mortality or resource use.^16^

### Estimation of hazard rates and HRs

Flexible parametric models (FPM) were used to model mortality in the cohort.^18,19^ A FPM can be used to estimate HRs, similarly to Cox regression, but, unlike the Cox model, it also estimates the baseline hazard rate using restricted cubic splines. Two FPMs were fitted first with all deaths considered as events, and secondly with death from external causes (ICD codes V01–Y98) as events, censoring at other deaths. Patients were followed from the index date until death and censored at emigration, end of study, or if any of the exclusion criteria were fulfilled. The exposure of interest, TRD, was treated as a time-varying covariate where a patient moved to the TRD group (i.e., from unexposed to exposed group) when the criteria for TRD were fulfilled. The two FPMs also adjusted for age at index date (using splines), sex, previous self-harm, previous substance use disorder, previous depression, other psychiatric comorbidity, and CCI (categorized as 0, 1, ⩾2 in the model for death due to external causes), and all of these were allowed to have time-varying effects (non-proportional hazards). Interactions between TRD and age (in categories 18–29, 30–49, 50–69), as well as TRD and CCI were included, since those were the interaction effects shown to be important in the previous study.^9^ Both FPMs had four degrees of freedom for modelling the baseline and two for time-varying effects.

### Estimation of standardized survival and excess deaths

Based on a fitted FPM, the survival probability at a certain point in time can be estimated for each patient, given the values of all variables included in the model for that specific patient. By taking the average of the estimated survival probabilities across all patients, a marginal survival probability is estimated, internally standardized to the covariate distribution within the cohort. Marginal all-cause 5-year survival was estimated for the group that developed TRD, separately for each combination of age group and CCI category. Within the cohort, TRD is a time-varying exposure; however, in the estimation of marginal survival, TRD-status was assumed to be fixed, so the survival was estimated as if everyone was TRD from start of and throughout 5 years of follow up. In the actual cohort, not all patients had 5 years of potential follow up, and even those who did, might not have 5 years of potential follow up as TRD exposed since TRD varies over time.

When interest lies in comparing survival between groups, while adjusting for other covariates, standardized survival can be used, by standardizing over the same covariate distribution for all groups. The average 5-year survival was therefore also estimated for non-TRD, separately for each combination of age group and CCI category, but standardized to the covariate distribution of the TRD patients. Since the only difference between the two standardized estimates within each age group and CCI category is the value set for the exposure of interest, the standardized survival estimates are comparable in terms of the confounder variables included in the model, and can be viewed as adjusted survival estimates.

Based on the 5-year standardized survival estimates, the expected number of deaths within 5 years was estimated, within each age group and CCI category. We then estimated the difference in number of expected deaths within 5 years for each age and CCI group combination, from the two standardized survival estimates. This can be interpreted as the number of deaths that could be avoided within 5 years among TRD patients, if they had the same mortality rate as patients with other depression (given their covariate distribution), within that specific age and CCI group. To aid comparison across age and CCI groups, we also estimated the number of expected deaths within 5 years for 1000 patients of each group. However, for each age and CCI group, the marginal/standardized survival was estimated given the distribution of other covariates within each of the groups. Differences in the number of expected deaths, between age and CCI groups, can therefore be due, to some extent, to differences in the distribution of all other factors taken into account in the analysis. The comparison between TRD and non-TRD is, however, due to the standardization, adjusted for all covariates. Standardized survival and number of excess deaths were also estimated for deaths due to external causes, using the same approach as described above.

### Ethical permission

The study was approved by the regional ethical review board in Stockholm (no. 2017/1236-31/2).

## Results

The depression cohort included 118,774 patients with a mean age of 37 (±14) years (58.2% women). Of these, 15,013 patients (13%) were classified as having TRD during the study period, with a mean age of 39 (±14) years (57.6% women). Characteristics of the population of patients with depression and TRD are shown in Table 1.

**Table 1.** Characteristics of the study cohort with depression and the subset of patients with TRD.

Age, sex, and history of depression and comorbidities	Entire cohort with depression			Proportion of the cohort with TRD		
	N (proportion of all) (%)	Deaths from all causes	Deaths from external causes (proportion of all causes) (%)	N (proportion of all) (%)	Deaths from all causes	Deaths from external causes (proportion of all causes) (%)
All	118,774 (100)	2727	1061 (38.9)	15,013 (100)	432	214 (55.8)
Age						
18–29 years	43,490 (36.6)	361	310 (85.9)	4820 (32.1)	66	58 (87.9)
30–49 years	48,373 (40.7)	726	383 (52.8)	6287 (41.9)	115	75 (65.2)
50–69 years	26,911 (22.7)	1640	368 (22.4)	3906 (26.0)	251	81 (32.3)
Sex						
Males	49,674 (41.8)	1659	712 (42.9)	6368 (42.4)	271	138 (50.9)
Females	69,100 (58.2)	1068	349 (32.7)	8645 (57.6)	161	76 (47.2)
History of depression^a^						
No	77,067 (66.9)	1500	543 (36.2)	9144 (60.9)	221	102 (46.2)
Yes <5 years ago	38,132 (32.1)	1136	477 (42.0)	5386 (35.9)	202	105 (52.0)
Yes ⩾5 years ago	3575 (3.0)	91	41 (45.1)	483 (3.2)	9	7 (77.8)
History of self-harm^a^						
No	111,537 (93.9)	2399	851 (35.5)	13,923 (93)	370	175 (47.3)
Yes	7237 (6.1)	328	210 (64.0)	1090 (7.0)	62	39 (62.9)
History of substance use disorder^a^						
No	107,160 (90.2)	2104	762 (36.2)	13,449 (89.6)	343	163 (47.5)
Yes	11,614 (9.8)	623	299 (48.0)	1564 (10.4)	89	51 (57.3)
History of other psychiatric comorbidity^a,b^						
No	91,972 (77.4)	2138	771 (36.1)	11,046 (73.6)	324	152 (46.9)
Yes	26,802 (22.6)	589	290 (49.2)	3967 (26.4)	108	62 (57.4)
CCIa						
0	103,854 (87.4)	1604	875 (54.6)	12,957 (86.3)	274	177 (64.6)
1	11,180 (9.4)	522	137 (26.3)	1593 (10.6)	88	30 (34.1)
>2	3740 (3.2)	601	49 (15.9)	463 (3.1)	70	7 (15.6)

a In the preceding 5 years.

b Anxiety disorder, attention deficit hyperactivity disorder, autism spectrum disorder, eating disorder, and personality disorder.

CCI, Charlson comorbidity index; TRD, treatment resistant depression.

Total follow-up time was 489,488 person years (mean 4.12 years). The amount of follow-up time as TRD was 54,697 years (mean 3.64 years) and with other depression 434,792 years (mean 3.78 years). The number of deaths during TRD-exposed follow-up time was 432, with 2295 during other depression follow-up time. During TRD follow-up time, 214 deaths (50%) were due to external causes (including 143 suicides), while during follow-up time with other depression, 849 (37%) deaths were due to external causes (including 550 suicides).

The number of TRD patients in each age- and CCI group is presented in Table 2, which also shows HRs for all-cause mortality in TRD compared with other depression, standardized/marginal 5-year survival, expected number of deaths, and numbers of estimated excess deaths. The HRs were highest for the youngest age group and the group with no comorbidities, which were the groups with highest survival. The marginal 5-year survival in TRD ranged from 98.0% (95% CI 97.6; 98.5), for the youngest group without comorbidities, to 74.4% (95% CI 69.6; 79.5) for the oldest group with a CCI of 2 or above, respectively. If the TRD patients had the same mortality rate as patients with other depression (otherwise given the same covariate distribution), this range for 5-year survival was found to be slightly higher: 99.1 (95% CI 99.0; 99.2) to 72.8 (95% CI 70.9; 74.7).

**Table 2.** HRs for all-cause mortality, expected number of deaths, and excess deaths for patients with TRD compared with other patients with depression.

	HR of all-cause mortality for TRD versus other depression (95% CI)	Number of patients with TRD	Standardized/marginal 5-year all-cause survival for TRD group if all were TRD from start (95% CI)	Standardized 5-year all-cause survival for TRD group if they had the survival of patients with other depression (95% CI)	Expected number of deaths within 5 years for TRD group if all were TRD from start (95% CI)	Expected number of deaths within 5 years for TRD group if they had the survival of patients with other depression (95% CI)	Excess deaths within 5 years among all patients with TRD (95% CI)	Expected number of deaths within 5 years per 1000 TRD patients if all were TRD from start (95% CI)	Expected number of deaths within 5 years per 1000 TRD patients with survival of patients with other depression (95% CI)	Excess number of deaths within 5 years per 1000 TRD patients (95% CI)
Age 18–29 years										
CCI 0	2.08 (1.59; 2.72)	4457	98.0 (97.6; 98.5)	99.1 (99.0; 99.2)	87 (66; 108)	42 (38; 47)	45 (24; 67)	20 (15; 24)	10 (8; 10)	10 (5; 15)
CCI 1	1.84 (1.29; 2.62)	326	96.3 (95.2; 97.5)	98.0 (97.7; 98.3)	12 (8; 16)	7 (6; 7)	5 (2; 9)	37 (25; 48)	20 (17; 23)	16 (5; 28)
CCI ⩾ 2	1.45 (0.99; 2.13)	37	92.6 (90.1; 95.1)	94.8 (94.0; 95.6)	3 (2; 4)	2 (2; 2)	1 (0; 2)	75 (49; 99)	52 (44; 60)	22 (−4;49)
Age 30–49 years										
CCI 0	1.57 (1.28; 1.93)	5577	97.9 (97.6; 98.3)	98.7 (98.6; 98.8)	115 (93; 136)	73 (68; 79)	41 (19; 63)	21 (17; 24)	13 (12; 14)	7 (3; 11)
CCI 1	1.39 (1.03; 1.87)	569	95.9 (94.8; 97.0)	97.0 (96.7; 97.4)	23 (17; 30)	17 (15; 19)	6 (0; 13)	41 (30; 52)	30 (26; 33)	11 (0; 23)
CCI ⩾ 2	1.09 (0.79; 1.51)	141	90.6 (88.0; 93.3)	91.3 (90.4; 92.3)	13 (10; 17)	12 (11; 14)	1 (−3; 5)	94 (67; 120)	87 (77; 96)	8 (−20; 35)
Age 50–69 years										
CCI 0	1.33 (1.12; 1.58)	2923	94.7 (93.8; 95.5)	95.9 (95.7; 96.2)	156 (132; 180)	119 (110; 127)	37 (12; 63)	53 (45; 62)	41 (38; 43)	13 (4; 21)
CCI 1	1.17 (0.92; 1.49)	698	89.1 (86.9; 91.3)	90.6 (89.7; 91.4)	76 (61; 91)	66 (60; 72)	10 (−6; 27)	109 (87; 131)	94 (86; 103)	15 (−8; 38)
CCI ⩾ 2	0.93 (0.72; 1.19)	285	74.4 (69.6; 79.5)	72.8 (70.9; 74.7)	73 (58; 87)	78 (72; 83)	−5 (−20; 10)	256 (205; 304)	272 (253; 291)	−16 (−69; 36)

CCI, Charlson comorbidity index; CI, confidence interval; HR, hazard ratio; TRD, treatment resistant depression.

Patients with TRD displayed excess deaths in all age and comorbidity categories where numbers were large enough for statistical significance. The largest number of expected all-cause excess deaths within 5 years was found among patients 18–29 years old with CCI 0, where 45 (95% CI 24; 67) of a total of 87 (95% CI 66; 108) TRD deaths were deemed excess deaths. Translating these numbers into excess deaths within 5 years per 1000 patients showed point estimates ranging from 7 to 16 in the age- and comorbidity groups with statistical significance. The highest significant point estimate was found in the age group 18–29 years with CCI 1, with 16 (95% CI 5; 28) of the expected 37 (95% CI 25; 48) TRD deaths per 1000 patients being excess deaths. There were non-significant estimates in the number of expected all-cause deaths in the age groups of 18–29 and 30–49 years with CCI 2 and above, and for the 50–69 age group with CCI 1 or 2 and above.

In Table 3, the same estimates as above are presented for deaths from external causes only. Again, the highest HRs and absolute numbers of estimated deaths were seen in the numerically largest patient categories. Numbers of estimated excess deaths from external causes were similar to estimates for all-cause deaths among younger and physically healthy patients – for instance, the same estimate, 10 deaths (95% CI 5; 15) per 1000 patients within 5 years was found among patients 18–29 years old with CCI 0 in both analyses – suggesting that the absolute majority of all expected deaths among these patients were from external causes. When estimating the numbers of excess deaths from external causes, the numbers of significant excess deaths were of a similar magnitude as for all-cause deaths, ranging from 9 to 15 per 1000 patients with TRD survival within 5 years. The largest number was estimated at 15 (95% CI 2; 28) of a total of 34 (95% CI 22; 47) expected TRD deaths in the age group 50–69 with CCI 1. For patients with CCI 2 and above, the point estimates for excess deaths were lower, but statistically non-significant.

**Table 3.** HRs for mortality due to external causes^a^, expected number of deaths, and excess deaths for patients with TRD compared with other patients with depression.

	HR of cause-specific mortality for TRD versus other depression (95% CI)^a^	Number of patients with TRD	Standardized/marginal 5-year cause-specific survival for TRD group if all were TRD from start (95% CI)^a^	Standardized 5-year cause-specific survival for TRD group if they had the survival of patients with other depression (95% CI)^a^	Expected number of deaths within 5 years for TRD group if all were TRD from start (95% CI)	Expected number of deaths within 5 years for TRD group if they had the survival of patients with other depression (95% CI)	Excess deaths within 5 years among all patients with TRD (95% CI)	Expected number of deaths within 5 years per 1000 TRD patients if all were TRD from start (95% CI)	Expected number of deaths within 5 years per 1000 TRD patients if they had the survival of patients with other depression (95% CI)	Excess deaths within 5 years per 1000 TRD patients (95% CI)
Age 18–29 years										
CCI 0	2.09 (1.57; 2.76)	4457	98.1 (97.6; 98.6)	99.1 (99.0; 99.2)	84 (63; 105)	41 (36; 46)	43 (21; 65)	19 (14; 27)	9 (8; 10)	10 (5; 15)
CCI 1	1.92 (1.17; 3.17)	326	97.8 (96.9; 98.8)	98.9 (98.6; 99.1)	7 (4; 10)	4 (3; 5)	3 (0; 7)	22 (12; 31)	11 (9; 14)	10 (1; 20)
CCI ⩾ 2	1.35 (0.57; 3.22)	37	98.7 (97.7; 99.7)	99.1 (98.7; 99.4)	0 (0; 1)	0 (0; 0)	0 (0; 1)	13 (3; 23)	9 (6; 13)	3 (−7; 14)
Age 30–49 years										
CCI 0	2.02 (1.56; 2.61)	5577	98.4 (98.0; 98.7)	99.2 (99.1; 99.3)	92 (71; 165)	46 (41; 51)	46 (24; 67)	16 (13; 20)	8 (7; 9)	8 (4; 12)
CCI 1	1.85 (1.16; 2.97)	569	98.1 (97.3; 98.9)	98.9 (98.7; 99.2)	11 (6; 16)	6 (5; 7)	5 (0; 10)	19 (11; 28)	11 (8; 13)	9 (1; 17)
CCI ⩾ 2	1.30 (0.56; 3.03)	141	98.3 (97.0; 99.6)	98.7 (98.3; 99.1)	3 (1; 4)	2 (1; 2)	1 (−1; 2)	17 (4; 30)	13 (9; 17)	4 (−10; 17)
Age 50–69 years										
CCI 0	1.99 (1.52; 2.61)	2923	97.3 (96.7; 97.9)	98.6 (98.5; 98.8)	79 (60; 97)	40 (35; 45)	39 (20; 58)	27 (21; 33)	14 (12; 15)	13 (7; 20)
CCI 1	1.83 (1.19; 2.81)	698	96.6 (95.3; 97.8)	98.1 (97.7; 98.5)	24 (5; 33)	13 (11; 16)	11 (2; 20)	34 (22; 47)	19 (15; 23)	15 (2; 28)
CCI ⩾ 2	1.29 (0.57; 2.89)	285	97.3 (95.3; 99.3)	97.8 (97.2; 98.5)	8 (2; 16)	6 (4; 8)	2 (-4; 8)	27 (7; 47)	22 (15; 28)	6 (−15; 27)

a Deaths from external causes, i.e., suicides and accidents (ICD-10 codes V01–Y98).

CCI, Charlson comorbidity index; CI, confidence interval; HR, hazard ratio; TRD, treatment resistant depression.

## Discussion

This is the first study to translate the increased mortality in TRD compared with other depression into estimated numbers of excess deaths. Excess deaths were found in most age and comorbidity categories, ranging from 7 to 16 deaths per 1000 patients with TRD during 5 years. Numbers of excess deaths from external causes, i.e., suicides or accidents, were similar to numbers of excess all-cause deaths, implying that the majority of excess deaths in TRD are due to external causes. The results illustrate how excess deaths can be used to expand and complement the previously reported increased HRs for mortality, standardized mortality ratios, and excess mortality rate ratios among patients with TRD.^9,20,21^

### Interpretation

The interactions of mortality with age and physical comorbidity were investigated specifically in this study. Within each age category, most point estimates for all-cause excess deaths were somewhat higher among patients with comorbidities (CCI ⩾1) compared with patients without comorbidities. This higher number of excess deaths could, if accurate, be attributed to several factors. First, patients with somatic comorbidity may be at higher risk for developing TRD compared with other patients, although evidence in the literature for this association is conflicting.^22^ It is also possible that TRD itself may have a negative impact on the course of somatic disease, and therefore contribute to the excess deaths among these patients. Studies have reported an increased mortality after acute myocardial infarction among patients with TRD compared with other depression,^23^ and persistent depressive symptoms may increase mortality after somatic hospitalization.^24^ Also, the construction of the CCI means that patients with different severity of the same somatic disease are classified with an equal score.^16^ This may leave residual confounding as severely ill patients with depression may be at higher risk for both TRD and death.^22^

In the analysis including only deaths from external causes, no clear pattern regarding somatic comorbidity could be seen, apart from lower point estimates for excess deaths among patient with major comorbidities (CC1 ⩾2). This may be due to suicide and somatic disease being competing risks of death for these patients. Regarding the interaction with age, however, both suicide risk and actual numbers of suicide are known to increase with age in the general population.^10^ In the present study, there were also slightly higher – albeit non-significant – numbers of excess deaths from external causes in 5 years per 1000 patients in the oldest age segment. However, as this was also the smallest group in numbers, absolute numbers of excess deaths were higher in the two younger age categories. Together, the investigation of these interactions emphasizes the importance of applying different measures of mortality in a population and identifying subgroups at risk, as patients who are in higher risk categories may not impact the overall numbers if numerically small.

### Implications

Previous research has established that the all-cause mortality among patients with depression is markedly higher than in the general population, and that this is even more pronounced for suicide.^25^ The results of the present study regarding excess deaths in TRD may be of both clinical and societal importance. Rates of TRD among patients initiating treatment for depression have been estimated to be 6%–29% in administrative data studies,^5,6^ and as high as 30%–50% in clinical studies.^3,4^ Considering that life-time risk for depression in the general population has been estimated at 15%–20%,^26^ the number of excess deaths in TRD is likely to be numerically impactful when transferring these rates to larger populations. According to the estimates in this study, TRD patients may experience up to twice as many deaths than if they had the survival of other patients with depression. Especially worrisome may be the estimated excess deaths among young, somatically healthy, patients, of which the large majority was due to external causes.

The patients with TRD in this study have all been administered several treatment trials for depression, as a proxy for depression that have not responded adequately to treatment, which may be a contributing factor to the excess deaths reported in this study. However, the group classified with TRD may be heterogeneous, as a substantial number of patients who present with TRD may have been misdiagnosed with depression and/or suffer from other undiagnosed psychiatric comorbidities,^27,28^ which could lead to worse antidepressant treatment results and may contribute to the excess deaths seen here. The TRD rate of 13% found in this cohort is substantially lower than the 30%–50% found in clinical studies,^3,4^ but is similar to other epidemiological studies in countries with comparable national databases (Denmark 14%, Taiwan 21%),^29,30^ and also to using other algorithms for defining TRD in Swedish data (9%–19%).^31^ The lower number of patients with TRD identified in observational studies compared with clinical studies may represent a lower tendency in real-life practice to administer more than two treatment trials, and some evidence points to the view that the smaller the proportion of patients identified with TRD, the higher the morbidity and poorer outcomes among patients.^31^

Regardless of the underlying mechanism, TRD as defined in this study – starting a third treatment trial for depression – seems to increase the risk for mortality and the number of deaths.

### Strengths and weaknesses

Strengths of this study include the cohort design with use of nationwide registers of high quality and virtually no loss of follow up, allowing for the calculation of survival rates in a large population for estimation of excess deaths. Among the limitations of this study is the use of a pharmacoepidemiological model for identifying TRD without access to clinical data aside from registered diagnoses. This lack of information includes unknown reasons for continuation, discontinuation or change of treatment trials (e.g., efficacy, non-efficacy, side effects, or lack of adherence). Patients over 70 years of age were not included due to hypothized competing risks between age-related deaths and deaths from sickness and external causes, and also due to likely differences in antidepressant treatment patterns compared with younger patients. Also, due to diagnoses registered in Swedish primary care not being included in the NPR, only patients seeking specialized psychiatric care were included. This may have attenuated the risk differences in the group comparison, for example, not including patients with milder or easily treated forms of depression. Finally, the PDR contains data from July 2005 onwards, which means that patients with TRD status before this date may have died before or during follow up without study inclusion.

## Conclusion

Patients with TRD experience all-cause excess deaths in the range of 7–16 deaths per 1000 patients during 5 years compared with if they had similar mortality as other patients with depression. More knowledge is needed regarding to what extent more effective treatments for depression may lower the number of excess deaths.

## Supplemental Material

sj-docx-1-tpp-10.1177_20451253211006508 – Supplemental material for Excess deaths in treatment-resistant depression Click here for additional data file. Supplemental material, sj-docx-1-tpp-10.1177_20451253211006508 for Excess deaths in treatment-resistant depression by Philip Brenner, Johan Reutfors, Michel Nijs and Therese M-L Andersson in Therapeutic Advances in Psychopharmacology
