# Supplementary material for: Excess deaths in treatment-resistant depression
Source: Ther Adv Psychopharmacol. 2021 Apr 12;11:20451253211006508. doi: 10.1177/20451253211006508 (PMC8047832; doi:10.1177/20451253211006508)
Supplement: sj-docx-1-tpp-10.1177_20451253211006508 – Supplemental material for Excess deaths in treatment-resistant depression [file sj-docx-1-tpp-10.1177_20451253211006508.docx]

**Supplemental Table 1.** Distribution of types of third treatment trial for meeting criteria for treatment resistant depression

|  | n | % |
| --- | --- | --- |
| Electroconvulsive therapy | 283 | 1.9 |
| Repetitive Transcranial Magnetic Stimulation | 0 | 0 |
| Mood stabilizer^1^ | 761 | 5.0 |
| Antipsychotic^2^ | 2387 | 15.8 |
| Lithium^3^ | 125 | 0.8 |
| Antidepressant^4^ | 11564 | 76.5 |
|  |  |  |
| Total | 15120 | 100.0 |

^1^Valproate (ATC code N03AG01), lamotrigine (N03AX09) and carbamazepine (N03AF01)
^2^ATC code N05A, not including N05AN01
^3^ATC code N05AN01
^4^ATC code N06A
